# Prediction of excessively low vault after implantable collamer lens implantation using iris morphology

**DOI:** 10.3389/fmed.2022.1029350

**Published:** 2022-10-28

**Authors:** Muhammad Ahmad Khan, Qian Tan, Wei Sun, Wang Cai, Libei Zhao, Ding Lin

**Affiliations:** ^1^Aier School of Ophthalmology, Central South University, Changsha, China; ^2^Department of Ophthalmology, Changsha Aier Eye Hospital, Changsha, China

**Keywords:** iris concavity, vault, implantable collamer lens, myopia, AS-OCT

## Abstract

**Purpose:**

To identify the iris morphology-related factors for prediction of outcomes of excessively low vault (< 100 μm) after Implantable Collamer Lens V4c (ICL V4c; STAAR Surgical) implantation.

**Methods:**

This retrospective case-control study included 81 eyes from 2,080 patients who underwent ICL implantation. Twenty-seven eyes of 27 patients with excessively low vault (< 100 μm) constituted the case group (excessively low vault group). Patients with vault (250 to 750 μm) were selected as the optimal vault group by matching anterior chamber depth, white-to-white distance and ICL size with cases with excessive low vault (< 100 μm) at a proportion of 1:2. The preoperative biometric parameters and postoperative vault were recorded. Multiple linear regression analysis was performed to assess the relationship between the postoperative vault and various variables. Conditional logistic regression analysis was used to estimate the risk factors for excessively low vault.

**Results:**

The postoperative vault was associated with preoperative pupil diameter (PD), crystalline lens rise, iris concavity and the ratio of the iris concavity to chord length (*P* < 0.05). The larger iris concavity increased risk of excessively low postoperative vault (< 100 μm) (OR = 81.10; 95%CI = 2.87 to 2296.58; *P* = 0.01).

**Conclusions:**

Eyes with obviously concave iris were associated with a higher rate of excessively low vault (< 100 μm). Evaluation of iris morphology may provide significant information for predicting excessive postoperative vault.

## Introduction

Myopia is a major cause of distance vision impairment, which has emerged as an increasingly prevalent public health problem worldwide (1). Particularly, myopes with high myopia and extremely high myopia are susceptible to be undercorrected. Despite the widely use of laser corneal refractive surgery to correct myopia, the amount of correction is limited for thin corneas, abnormalities in their corneal morphology manifested in topography, or high ametropia.

The Visual Implantable Collamer Lens (ICL; STAAR Surgical, Nidau, Switzerland), a posterior chamber phakic intraocular lens, is popularly used for the correction of high myopia. It can eliminate the risk of corneal ectasia as compared with laser corneal refractive surgery and provide satisfactory visual outcome (2). However, the safety of ICL implantation is dependent on accurate ICL size and appropriate vault. Excessively high vault can be associated with pupillary block, angle closure, pigment dispersion and subsequent glaucoma. The Visian V4c ICL with a 0.36 mm central port (KS-Aquaport) allows the physiological flow of aqueous humor, which can prevent pupillary block and decrease the risk of elevated intraocular pressure (3). On the other hand, excessively low vault is regarded as one risk factor of anterior subcapsular cataract. As reported, the minimum required central vault to avoid the incidence of cataract varied from 150 to 230 μm (4, 5) and subsequent ICL exchange was performed when insufficient vault was < 100 μm (6). Although the Visian V4c ICL with a central hole can maintain crystalline lens epithelium cells metabolism which may reduce the incidence of cataract, the consequences of the contact of ICL with the crystalline lens remain to be major concern.

Accurate prediction of the vault remains challenging due to the difficulties in preoperative estimation of a relationship between the anatomical structures of the eye and ICL. Recent studies demonstrated that insufficient vault was related to high protrusion of crystalline lens, wide sulcus-to-sulcus, flat cornea and low myopic ICL power (7, 8). Despite the variety of associations put forward, excessively low vault still occurs in actual clinical settings, and sometimes ICL exchange is required to avoid low vault related complications. Previous study found that an anterior-posterior compression caused by the iris leading to lower vault (9). Considering the ICL was placed behind the iris, the horizontal and vertical compression by the iris may influence the postoperative vault (10). It remains unclear whether the morphology of the iris was the predictive factor related to the excessively low vault.

With anterior segment optical coherence tomography (AS-OCT), preoperative biometric parameters (such as lens position, iris morphology and anterior chamber width) can be obtained rapidly through noncontact procedures. Therefore, the present study aimed to analyzed the shape of iris that may affect the achieved ICL vault, resulting in an excessively low vault (< 100 μm) after ICL implantation based on AS-OCT as compared with the expected vault, thereby facilitating the determination of appropriate ICL size to reduce the occurrence of excessively low vault and ICL exchange.

## Methods

### Patients

This study was approved by the Ethics Committee of the Changsha Aier Eye Hospital, Aier Eye Hospital Group, China (ID:2020KYYJ004), and all procedures were conducted according to the tenets of the Declaration of Helsinki. All participants were fully informed about the surgical details and provided written informed consent.

This is a retrospective study which includes 81 eyes from 2080 patients implanted Vision ICL V4c implantation to the correction of myopia at Changsha Aier Eye Hospital from January 2020 to February 2022. Patients were followed up on 1 day, 1 week, 1, 3, 6, and 12 months after surgery. To probe into factors exerting significant influence on excessively low vault, a case-control study was performed. Twenty-seven eyes of 27 patients with vault of < 100 μm on postoperative 1 month constituted the case group (excessively low vault group). And patients with vault of between 250 and 750 μm on postoperative 1 month were selected using a systematic sampling method by matching anterior chamber depth (ACD, ±0.2 mm), horizontal white-to-white distance (WTW, ±0.1 mm) and ICL size with cases with excessively low vault (< 100 μm) at a proportion of 1:2.

The inclusion criteria of this study were: (1) age ranging from 21 to 45 years; (2) spectacle spherical power between −0.50 and −18.00 diopters (D); cylindrical power lower than −5.00 D; (3) ACD ≥2.8 mm and corneal endothelial cell density >2,000 cells/mm^2^; (4) the implanted ICL sizing was determined strictly according to the manufacturer's recommendations. The exclusion criteria were as follows: preexisting ocular pathology, previous ocular surgery and chronic systemic diseases.

### Preoperative and postoperative examinations

Preoperative ocular examinations, including manifest refraction, non-contact tonometry, slit-lamp microscopy, examination of the fundus, and endothelial cell density measurement were conducted to all participants. WTW, central corneal thickness (CCT), pupil diameter (PD) and keratometry were measured using a Scheimpflug camera (Pentacam HR; Oculus Optikgerate GmbH).

**Figure 1:**
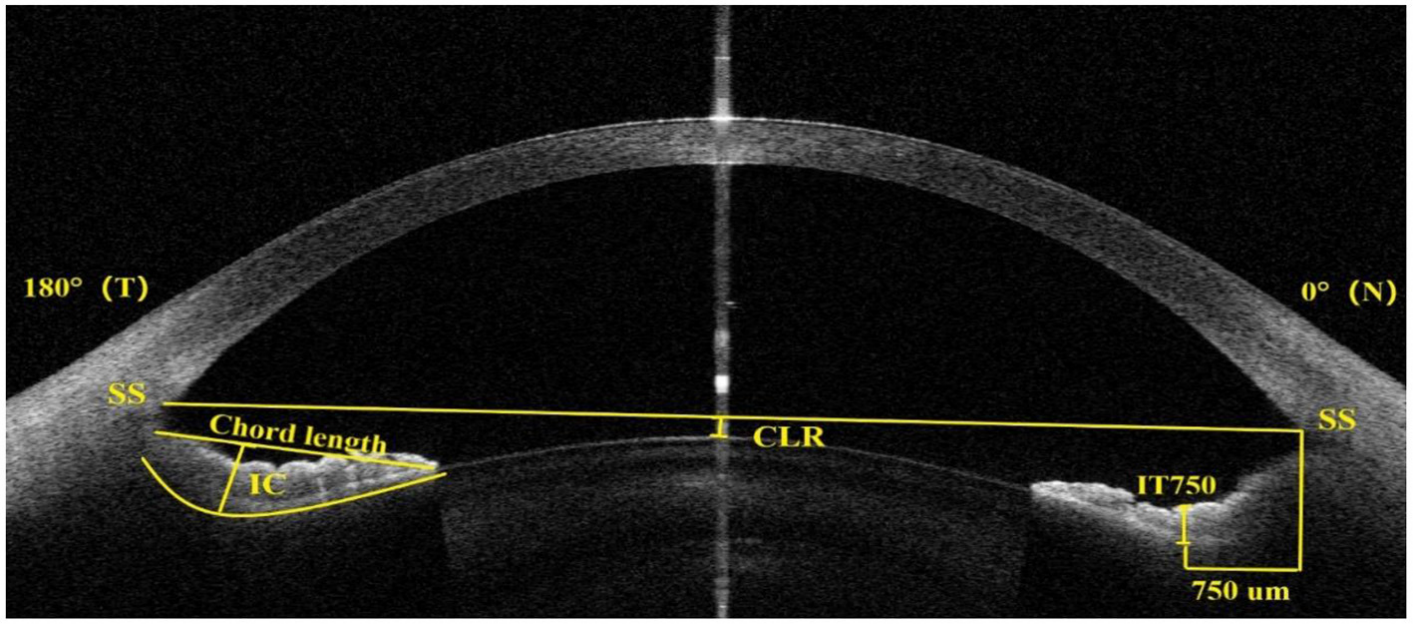
Anterior segment optical coherence tomography image showing the measurement of the crystalline lens rise (CLR), iris concavity (IC), the ratio of the iris concavity to chord length (ICR), iris thickness at 750 μm from the scleral spur (IT750) and scleral spur (SS).

Anterior segment imaging was performed using swept-source anterior segment optical coherence tomography (AS-OCT, CASIA2, Tomey corporation, Nagoya, Japan), as described previously (11, 12). The AS-OCT parameters were measured by horizontal scan across the pupil center, including ACD, anterior chamber width (ACW), crystalline lens rise (CLR), iris concavity (IC), iris concavity ratio (ICR), iris thickness at 750 μm from the scleral spur (IT750) and postoperative vault. The ACD was measured from the corneal endothelium at the corneal apex to the crystalline lens anterior surface. ACW was defined as the distance between the scleral spurs on the temporal and nasal and sides. As shown in Figure 1, CLR was the perpendicular distance between the lens anterior surface and a horizontal line joining the two scleral spurs. IC was defined as the maximum distance between the iris posterior surface and the chord from the iris root to the most peripheral point of contact between the iris and lens. ICR was defined as the ratio of the iris concavity to chord length. The ICL vault was measured by AS-OCT on postoperative 1 day, 1 week, and 1, 3, 6, and 12 months.

### Statistical analysis

Statistical analyses were performed with SPSS Statistics (version 25, IBM Corp., NY, USA). The normal distribution of the data was tested using the Shapiro–Wilk test. Independent sample *t*-test was applied to compared normally distributed continuous variables. Mann-Whitney *U*-test was used to analyze skewed continuous variables. Chi-square test was used to compare categorical variables. Multiple linear regression analysis was performed to assess the relationship between the postoperative vault and various variables. Conditional logistic regression analysis was subsequently used to estimate independent risk factors for the presence of postoperative vault less than 100 μm. A *P* < 0.05 indicated a statistically significant difference.

## Results

**Figure 2:**
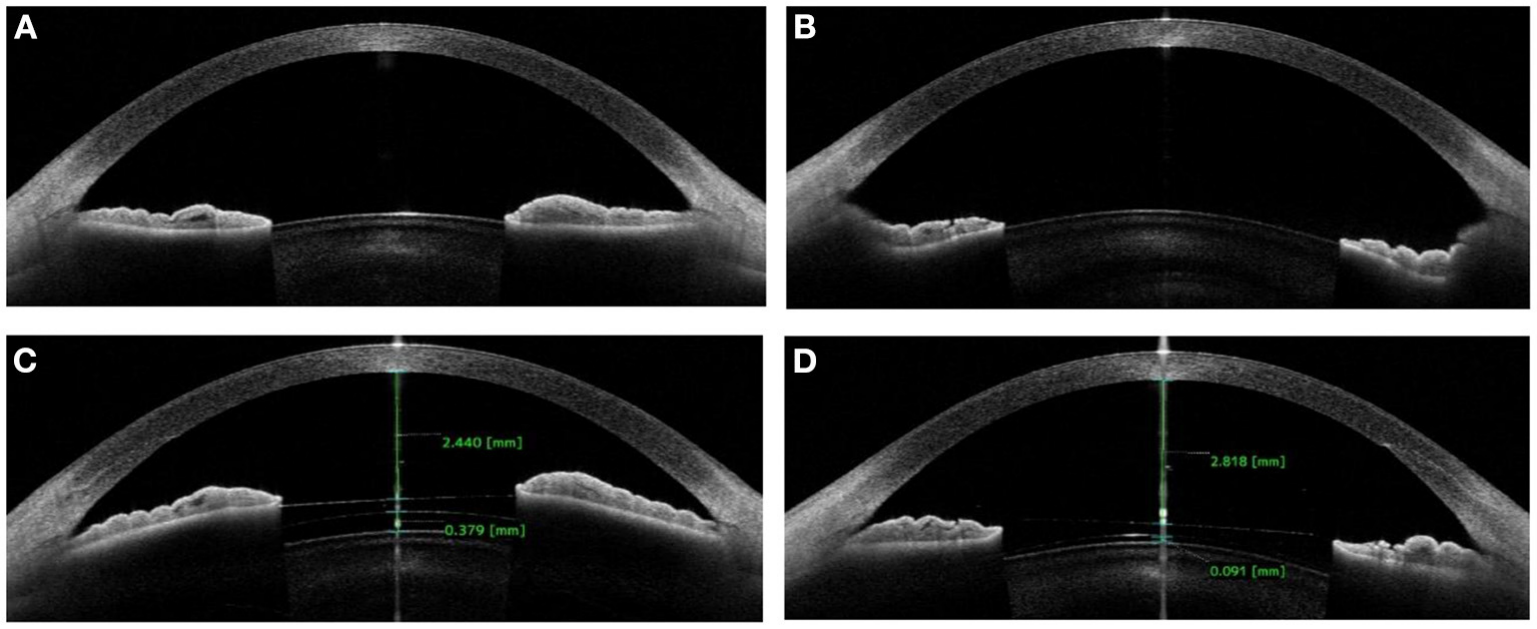
Anterior segment optical coherence tomography images showing flat iris **(A)** with acceptable postoperative vault **(C)** in normal group; and concave iris **(B)** with low vault **(D)** in excessively low vault group.

The demographic and ocular biometric characteristics for different vault groups are summarized in Table 1. Comparison AS-OCT images of exhibiting iris concavity with postoperative vault in different groups are presented in Figure 2. The excessively low vault group includes eyes with a vault of < 100 μm (27 eyes), whereas the normal vault group includes eyes with a vault between 250 and 750 μm (54 eyes). The mean postoperative vault was 78.85 ± 23.61 μm and 539.63 ± 120.82 μm, respectively, in the excessively low vault group and normal vault group. There were no significant differences between the two groups except for CLR, IC and ICR. Eyes in the excessively low vault group had a greater CLR compared with the normal vault group (*P* < 0.001). Among parameters related to the iris morphology measured by AS-OCT, eyes in the excessively low vault group had larger IC and ICR compared to the eyes in the normal vault group (*P* < 0.001).

**Table 1:** Comparisons of demographics and ocular biometric parameters of patients between the excessively low vault and normal vault groups.

**Parameter**	**Excessively low**	**Normal vault**	***P***
	**vault (*n* = 27)**	**(*n* = 54)**	
Age (years)	28.93 ± 5.76	28.93 ± 5.05	0.93^c^
Gender (M/F)	8/19	15/39	0.86^b^
SE (D)	−8.33 ± 2.64	−7.97 ± 2.15	0.35^c^
ACD (mm)	3.12 ± 0.10	3.15 ± 0.10	0.07^c^
IOP (mmHg)	14.87 ± 2.10	15.17 ± 1.92	0.53^a^
WTW (mm)	11.29 ± 0.33	11.29 ± 0.31	0.92^c^
ACW (mm)	11.61 ± 0.34	11.43 ± 0.34	0.34^a^
CCT (mm)	0.51 ± 0.03	0.51 ± 0.03	0.44^a^
Kf (D)	43.05 ± 1.34	42.55 ± 0.87	0.09^a^
Ks (D)	44.30 ± 1.58	43.63 ± 1.09	0.06^a^
PD (mm)	5.65 ± 1.04	5.72 ± 0.86	0.75^a^
CLR (mm)	−0.05 ± 0.17	−0.27 ± 0.09	< 0.001^a, d^
IT750 (mm)	0.42 ± 0.05	0.42 ± 0.04	0.70^c^
IC (mm)	0.24 ± 0.07	0.11 ± 0.04	< 0.001^c, d^
ICR (mm)	0.07 ± 0.02	0.03 ± 0.01	< 0.001^c, d^
Postoperative vault (μm)	78.85 ± 23.61	539.63 ± 120.82	< 0.001^c, d^

SE, spherical equivalent; D, diopters; ACD, anterior chamber depth; IOP, intraocular pressure; WTW, horizontal white-to-white diameter; ACW, anterior chamber width; CCT, central corneal thickness; Kf, flat keratometry; Ks, steep keratometry; PD, pupil diameter; CLR, crystalline lens rise; IT750, iris thickness at 750 μm from the scleral spur; IC, iris concavity; ICR, the ratio of the iris concavity to chord length.

a Independent t-test.

b Chi-square test.

c Mann-Whitney U test.

d *P* < 0.05.

The preoperative ocular biometric parameters were strictly selected in the regression model using simple linear regression. One variable was selected in the analysis if the correlation coefficient of the two variables was larger than 0.6. Finally, six parameters were selected in the multiple linear regression analysis (Table 2). The results of multiple linear regression analysis showed that the PD, CLR, IC and ICR were the relevant variables related to the vault after ICL implantation (*P* < 0.05).

**Table 2:** Results of multiple linear regression analysis evaluating the correlation between preoperative parameters and postoperative vault (*n* = 81).

**Variable**	**β**	***t***	***P***
ACW (mm)	0.18	1.67	0.10
PD (mm)	0.26	2.80	0.007^*^
CLR (mm)	−0.29	−3.07	0.003^*^
IT750 (mm)	−0.05	−0.73	0.47
IC (mm)	−1.10	−3.09	0.003^*^
ICR (mm)	0.76	2.27	0.026^*^

β, unstandardized coefficient; ACW, anterior chamber width; PD, pupil diameter; CLR, crystalline lens rise; IT750, iris thickness at 750 μm from the scleral spur; IC, iris concavity; ICR, the ratio of the iris concavity to chord length.

* *P* < 0.05.

Concerning the risk of developing excessively low vault < 100 μm, a multivariable conditional logistic regression model was performed to determine predictive risk factors (Table 3). The IC (OR = 81.10; 95%CI = 2.87 to 2296.58; *P* = 0.01) was found to be the only independent risk factors for prediction of excessively low postoperative vault.

**Table 3:** Results of conditional logistic regression analysis assessing the risk factors related to excessively low vault (< 100 μm) (*n* = 81).

**Variable**	**β**	**SE**	**OR (95%CI)**	***P***
ACW (mm)	0.38	0.53	1.46 (0.51 to 4.13)	0.48
PD (mm)	−0.18	0.52	0.84 (0.31 to 2.30)	0.73
CLR (mm)	−0.32	0.71	0.73 (0.18 to 2.91)	0.66
IT750 (mm)	−0.16	0.49	0.86 (0.33 to 2.22)	0.75
IC (mm)	4.40	1.71	81.10 (2.87 to 2296.58)	0.01^*^
ICR (mm)	0.26	0.50	1.29 (0.49 to 3.44)	0.61

β, regression coefficient; SE, standard error; OR, odds ratio; CI, confidence interval; ACW, anterior chamber width; PD, pupil diameter; CLR, crystalline lens rise; IT750, iris thickness at 750 μm from the scleral spur; IC, iris concavity; ICR, the ratio of the iris concavity to chord length.

* *P* < 0.05.

## Discussion

Accurate prediction of the postoperative vault remains challenging for refractive experts. Many studies have attempted to solve the problem by developing ICL sizing formulas and exploring more influencing factors. However, unexpected excessively low vault still occurs occasionally, leading to ICL exchange and cataract formation. It arouses more thinking on whether some existing underlying factors that have been ignored previously. A previous study by Kojima and associates, using high-frequency ultrasound biomicroscopy (UBM), showed that the distance between sulcus-to-sulcus plane and anterior crystalline lens surface (STSL) was defined as a predictor for optimal ICL sizing (13). UBM is invasive and shows poor reproducibility due to unstable measurement sites. In contrast, AS-OCT is already being widely used to develop ICL sizing formulas and predict postoperative vault, owing to its non-invasiveness and conduciveness to preoperative measurement and postoperative follow-up. Nakamura et al. (14) developed a novel ICL sizing method based on the AS-OCT, in which CLR proved to be a candidate for predicting the vault after ICL implantation. The influence of crystalline lens position on the postoperative vault has been reported in various studies (15, 16). As reported, the CLR represented the anterior portion of the lens (17). In the present study, higher CLR (−0.05 ± 0.17) were found in the excessively low vault group, whereas the normal vault group tended to have significantly lower mean values (−0.27 ± 0.09). Regression analysis also disclosed that CLR was associated with postoperative vault. Our findings were in agreement with previous studies that described a forward lens position or high crystalline lens rise could have lower postoperative vault. In addition, our study also showed that the PD was a relevant parameter related to the postoperative vault. Consistently, Gonzalez-Lopez et al. (18) observed that the changes in the pupil size under different light conditions were associated with the vault after ICL implantation based on dynamic AS-OCT. Myopic eyes with larger pupil size were often predisposed to higher postoperative vault (19). These findings indicated that the causative factor for the change in pupil size might be considered preoperatively.

AS-OCT parameters including IC and ICR were measured to assess the shape of iris. IC could better quantify the displacement of the iris. Consequently, iris concavity was selected to represent the morphology of the iris in the regression model. The results of regression analysis showed that IC and ICR influenced the postoperative vault. Additionally, the concavity of iris was the only independent risk factors for prediction of excessively low postoperative vault. This finding indicated that the eyes with larger iris concavity were associated with a higher rate of excessively low vault (< 100 μm) after ICL implantation. However, IT750 were not statistically correlated with the postoperative ICL vaulting. As the iris draped over the lens and acted as a reverse force, we speculated that the reverse force of the iris prevented the rise of vault after ICL implantation and concave iris could increase reverse force and finally induce excessively low vault. As known, concave iris configuration was associated with pigment dispersion syndrome due to increased iridolenticular contact (20). ICL implantation increases iridozonular and iridociliary-process distances, which may minimize iridolenticular contact. A recent study (21) investigated the influence of the iris and ciliary body morphology on the postoperative vault and indicated that eyes with a thicker lens and larger iris reverse convexity were significant correlation with the insufficient postoperative vault. Meanwhile, in our current study, all ICLs in the excessively low vault group were replaced for a larger lens sizing and obtained optimal postoperative vaults (range from 250 to 750 μm). Based on above conditions, we speculated that underestimation of the ICL size and consequently a risk of insufficient vault might happen when a conventional ICL sizing method was used in eyes with larger iris concavity. Thus, exchanging ICL for the larger size seemed a reasonable solution to avoid the occurrence of excessively low vault in these patients.

The results of this research highlight important aspects for influence on postoperative vault prediction. To the best of our knowledge, this is the first study attempting to evaluate the iris morphology-related factors for prediction of outcomes of excessively low vault (< 100 μm) after ICL implantation. On the basis of our findings, eyes with obviously concave iris were associated with a higher rate of excessively low vault after ICL implantation. Consequently, in addition to the lens position, the concavity of iris should be considered as an important factor in the selection of ICL sizing and the prediction of the postoperative vault.

The primary limitations of this study are the retrospective and observational nature of the study and the relatively small sample size. Furthermore, we only observed the horizontal iris morphology, absence of records of vertical iris morphology might restrain us figuring out their roles in affecting postoperative vault. A large prospective cohort study containing different directions of iris morphology is essential to confirm our findings in the future.

In conclusion, larger iris concavity was a major risk factor in eyes presenting excessively low vault. This finding offers a new insight about the preoperative strategy in ICL size and the prediction of postoperative vault.

## Data availability statement

The original contributions presented in the study are included in the article/supplementary material, further inquiries can be directed to the corresponding authors.

## Ethics statement

Written informed consent was obtained from the individual(s) for the publication of any potentially identifiable images or data included in this article.

## Author contributions

DL and LZ designed and directed the project. MK and LZ carried out the study and drafted the manuscript. QT, WS, and WC participated in collection, management, analysis, and interpretation of data. All authors were involved in review and final approval of the manuscript.

## Funding

This study was supported in part by the research project of the Science Research Foundation of Aier Eye Hospital Group (Grant No. AF2106D3).

## Conflict of interest

The authors declare that the research was conducted in the absence of any commercial or financial relationships that could be construed as a potential conflict of interest.

## Publisher's note

All claims expressed in this article are solely those of the authors and do not necessarily represent those of their affiliated organizations, or those of the publisher, the editors and the reviewers. Any product that may be evaluated in this article, or claim that may be made by its manufacturer, is not guaranteed or endorsed by the publisher.
